# Structural Characteristics of Reaction Tissue in Plants

**DOI:** 10.3390/plants12081705

**Published:** 2023-04-20

**Authors:** Litong Liu, Yu Luan, Changhua Fang, Jinbo Hu, Shanshan Chang, Benhua Fei

**Affiliations:** 1 College of Materials Science and Engineering, Central South University of Forestry and Technology, Changsha 410004, China; 2 International Centre for Bamboo and Rattan, Beijing 100102, Chinafeibenhua@icbr.ac.cn (B.F.); 3 Key Laboratory of National Forestry and Grassland Administration/Beijing for Bamboo & Rattan Science and Technology, Beijing 100102, China

**Keywords:** reaction tissue, growth stress, tension wood, compression wood, gelatinous fibers

## Abstract

To maintain or adjust posture under the challenges of gravity and increased self-weight, or the effects of light, snow, and slope, plants have the ability to develop a special type of tissue called reaction tissue. The formation of reaction tissue is a result of plant evolution and adaptation. The identification and study of plant reaction tissue are of great significance for understanding the systematics and evolution of plants, the processing and utilization of plant-based materials, and the exploration of new biomimetic materials and biological templates. Trees’ reaction tissues have been studied for many years, and recently, many new findings regarding these tissues have been reported. However, reaction tissue requires further detailed exploration, particularly due to their complex and diverse nature. Moreover, the reaction tissues in gymnosperms, vines, herbs, etc., which display unique biomechanical behavior, have also garnered the attention of research. After summarizing the existing literature, this paper provides an outline of the reaction tissues in woody plants and non-woody plants, and lays emphasis on alternations in the cell wall structure of the xylem in softwood and hardwood. The purpose of this paper is to provide a reference for the further exploration and study of reaction tissues with great diversity.

## 1. Introduction

During their growth processes, plants inevitably experience the effects of internal and external stimuli, such as gravity, intracellular turgor, light, wind, and snow [1], and respond with corresponding adaptations, which can affect the appearance, structure, and performance of their biology [2,3,4]. It is common for young trees to accidentally tilt due to the rotation of their root systems, which may be caused by strong winds or landslides. While they may be restored to a vertical position in the following years, such growth changes often result in unique eccentric growth in their bases [5]. The Monterey cypress, which often has an asymmetric appearance due to the influence of one-sided sea winds, is a typical example of this [6]. Another well known example is that after removing the apical shoots of some plants, the side shoots tend to bend upward and take over as the new leader through an internal mechanism, subsequently leading to the formation of a thickened flank [7]. The process of controlling a plant’s posture and preserving its shape in such cases is greatly assisted by alternations to the plant’s cell wall structure, which regulates mechanical stress as well as other features, such as its growth pattern and stiffness.

To maintain or adjust their shape and growth orientation, plant cells differentiate and adjust their structural features to form a special tissue called reaction tissue. Plant reaction tissue can be analogized to animal muscle [8,9,10,11]. During its formation, reaction tissue produces relatively high mechanical stress through variations in the plant’s cell wall structure, thereby exerting its biomechanical role. Plant reaction tissue is the result of the continuous evolution of plants during their growth, and its cell morphology, chemical composition, and mechanical properties differ from those of normal tissues. The study of plant reaction tissue is of great significance for exploring plant growth and development, and for the refinement and high-value utilization of plants. Reaction tissue in trees, especially reaction tissue in tree trunks, have been extensively studied, and researchers have explored its biomechanical function as well as its macroscopic and microscopic structural features. In recent years, there have been new discoveries regarding reaction tissues in other parts of trees and in other plants. This article summarizes and analyzes the types and structural features of reaction tissues in trees and other plants in order to provide a reference for further research in the fields of plant structure, function, material processing, and utilization.

## 2. Reaction Tissues in Woody Plants

Due to external factors, such as slope, wind, and forest edge effects, originally upright tree trunks may become tilted or bent. To withstand external forces and avoid being overwhelmed, woody plants form a non-normal tissue called reaction wood [12,13]. Studies have shown that reaction wood experiences abnormal growth stress compared with normal wood. Growth stress is a type of internal stress generated during the growth of trees and is the result of the interaction force between differentiated cells during their maturation [14]. Growth stress can be evaluated by measuring the micro-strain caused by stress release (the growth stress indicator value or the GSI value). The greater the absolute GSI value, the greater the growth stress [12]. The released strain can be converted into growth stress by using the elastic modulus collected from the position where the strain was measured. This strain is usually called growth strain.

**Figure 1:**
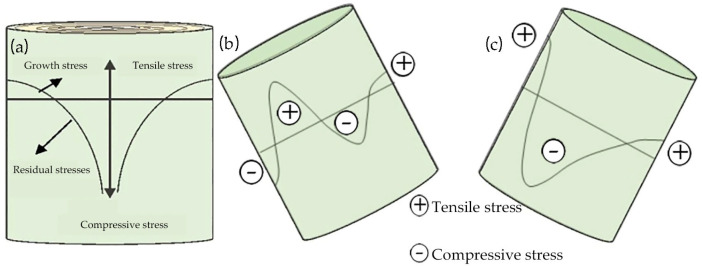
Distributions of longitudinal growth stress and residual stress from pith outwards in trunks [18]: (**a**) normal wood with symmetrical growth stress; (**b**) compression wood on the lower face with longitudinal compressive stress; (**c**) tension wood on the upper face with longitudinal tensile stress.

In normal upright tree trunks, growth stress is usually symmetrically distributed (Figure 1a). However, the formation of reaction wood results in greater tensile stress on the upper side of the leaning trunks and branches of hardwood species, and greater compressive stress on the lower side of the leaning trunks and branches of conifer trees. This asymmetric distribution of growth stress is key for trees to respond to external environmental forces and maintain or adjust their trunk and branch orientation [15,16,17].

**Figure 2:**
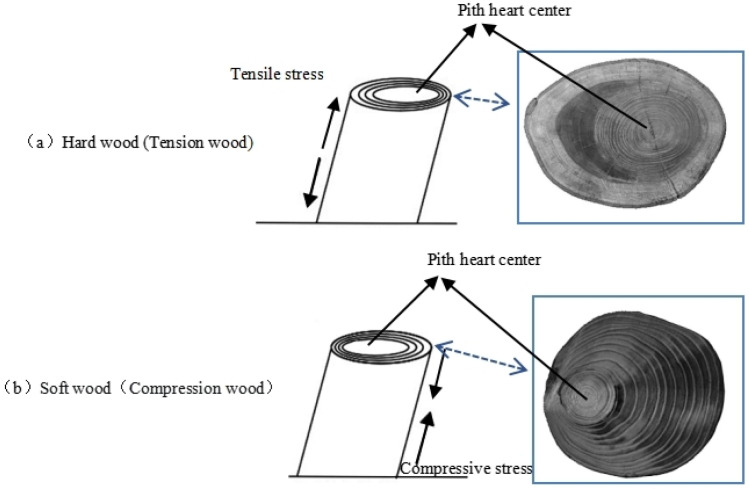
Eccentric growth of a stem related to the occurrence of (**a**) tension wood and (**b**) compression wood [22].

The formation of reaction wood is usually accompanied by eccentric growth (Figure 2), and observing eccentricity is one of the most intuitive methods for detecting the formation of reaction wood [19,20,21]. Generally, reaction wood in hardwood is located on the upper side of a tilted trunk and is called tension wood (Figure 2a); reaction wood in softwood is located on the lower side of a tilted trunk and is called compression wood (Figure 2b) [9,12].

### 2.1. Reaction Wood in Hardwood Species

The formation of tension wood in hardwood species usually occurs on the upper side of inclined stems or branches, with the pith being located on their lower side [23,24]. However, some tree species do not show obvious eccentricity, such as *Castanea sativa* [25] and *Bagassa guianensis* [26]. Some researchers have found that opposite eccentricity may occur when tension wood is formed [27] and have even observed tension wood on the lower side of trunks in some tree species [24,28]. Tension wood can also be produced in straight upright trunks that grow rapidly and have no eccentricity.

**Figure 3:**
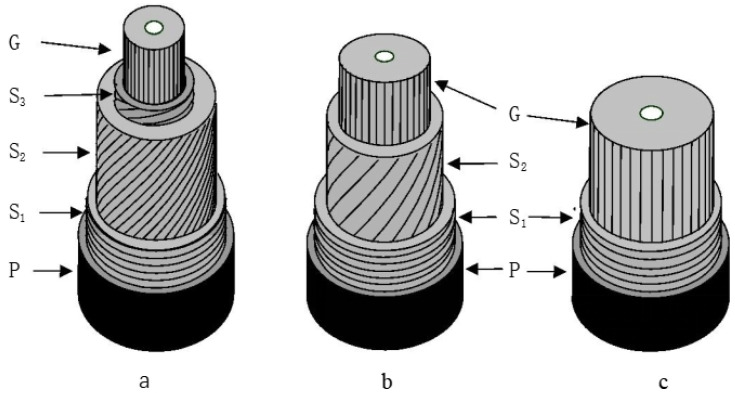
Cell wall structure of three types of gelatinous fibers: (**a**) a G-layer in addition to the three layers of the normal secondary cell wall; (**b**) a G-layer that replaces the S_3_ layer; (**c**) a G-layer that replaces both the S_2_ and S_3_ layers. P: primary wall; S_1_: outer layer of the secondary wall; S_2_: middle layer of the secondary wall; S_3_: inner layer of the secondary wall; G: gelatinous layer.

**Figure 4:**
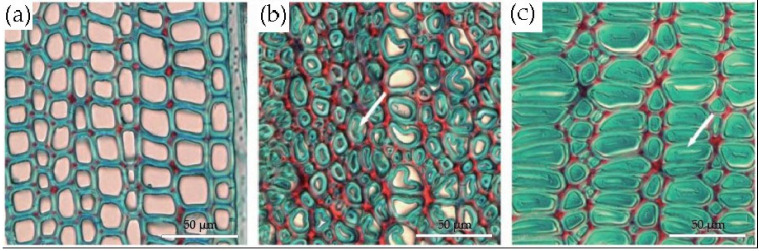
Transverse sections of three types of tropical tension wood stained with Safranin O/Fast Green [38] (arrows indicate the gelatinous layers): (**a**) Fiber cells of *Virola michelii* without gelatinous layers, which are similar to normal fiber cells; (**b**) gelatinous fibers of *Inga alba*, whose gelatinous layers’ thicknesses are similar to that of their adjacent layers; (**c**) gelatinous fibers of *Sextonia rubra* with thick gelatinous layers and negligible cavities.

At the microscopic level, the formation of tension wood is usually accompanied by the formation of a gelatinous layer (G-layer) in the cell wall [18,28]. The gelatinous layer is a manifestation of variation in the cell wall structure in response to external stimuli, and it can occur at different stages of the formation of secondary walls of cells [22,28]. Panshin and de Zeeuw [29] summarized the relevant literature and found that tension wood with gelatinous fibers usually has three types of wall layer structures, as shown in Figure 3: (1) a G-layer in addition to the three layers of the normal, lignified, secondary cell wall (Figure 3a); (2) a G-layer that replaces the S_3_ layer, with the S_1_ and S_2_ layers remaining (Figure 3b); (3) a thick G-layer that replaces both the S_2_ and S_3_ layers, with the S_1_ layer remaining (Figure 3c). The G-layer is mainly or entirely composed of cellulose, has a small amount of hemicellulose or other polysaccharide components and a small amount of lignin [23,30], and contains a large number of pores [31,32]. The microfibrils of the G-layer are almost parallel to the fiber axis [17,33], while the adjacent wall layers have a larger microfibrillar angle [34,35,36]. In microscopic observations, the G-layer can be distinguished from other wall layers based on the physical and chemical reactions of different staining agents with the cell wall of gelatinous fibers, using double staining techniques [37]. A G-layer with a very low lignin content is stained green with Fast Green or blue with Astral Blue, while lignified cell walls are stained red with Safranin (Figure 4).

**Figure 5:**
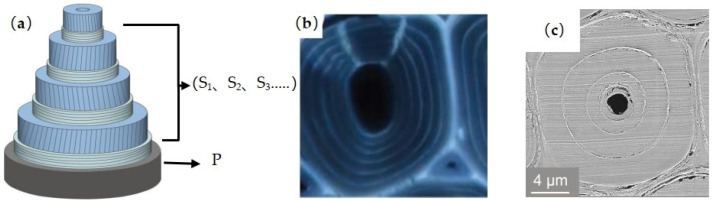
(**a**) Schematic diagram of tension wood fiber wall with multiple layers consisting of alternating broad and narrow layers; (**b**) *Laetia procera* tension wood fiber structure [46]; (**c**) *Phyllostachys pubescens* fiber structure. P: primary wall; S: secondary wall.

The G-layer is an important feature of tension wood, but it is not the only criterion for identifying tension wood [15,17]. The cell wall structure of tension wood has a rich diversity [38]. In addition to having fiber cells with G-layers, some tropical tree species or non-tropical primitive angiosperms (such as Liriodendron tulipifera Linn. in North America) often do not produce G-layers in their inclined trunk or branch fiber cells (Figure 4a), but their wood properties are similar to those of typical tension wood (i.e., tension wood with gelatinous fibers) [39,40,41,42,43]. For example, Clair et al. [44] measured the growth strain of 21 tropical tree species and found that only seven species (33% of the studied species) had obvious gelatinous fibers in their tension wood. Further, Onaka [28] found gelatinous fibers in only 40% of the tension wood of 346 Japanese tree species. In addition, some tension wood fiber cells exhibit a multilayer structure with alternating thick and thin layers (Figure 5a,b), and the number of layers in their secondary cell wall is positively correlated with the severity of the tension wood [20,21,45,46]. Moreover, the S_2_ layer of cells in the tension wood without G-layers also exhibits some structural features similar to G-layers, such as small microfibrillar angles, large cellulose crystallite sizes, and increased crystallinity [9,47,48].

### 2.2. Reaction Wood in Softwood Species

**Figure 6:**
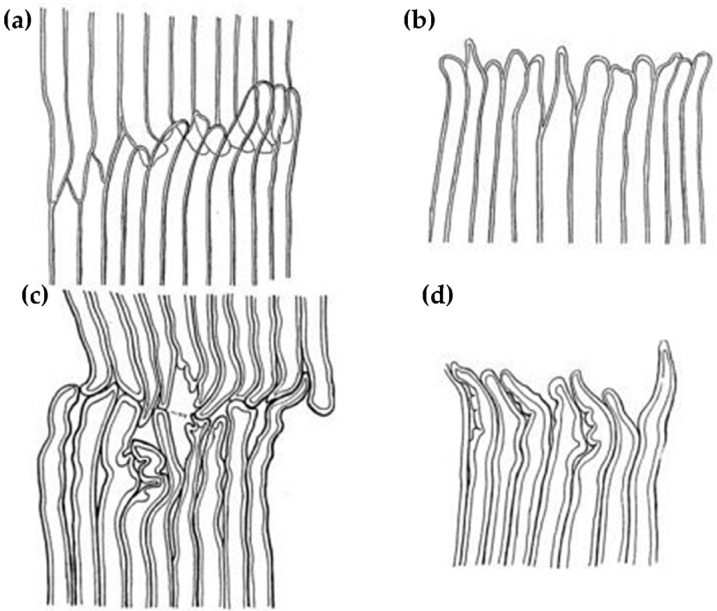
Normal wood tracheids (**a**,**b**) and compression wood tracheids (**c**,**d**) [53].

**Figure 7:**
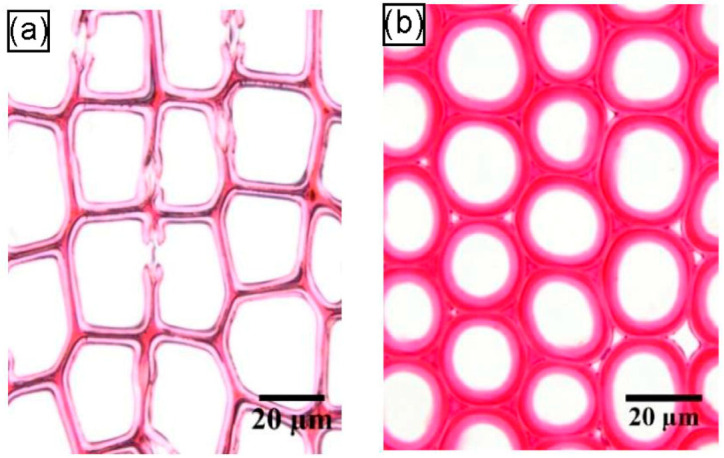
(**a**) Tracheids of Taxodium ‘*Zhongshanshan*’ normal wood with a square shape, whose cell corners and compound middle lamellae are stained dark red and secondary walls are stained light red; (**b**) tracheids of Taxodium ‘*Zhongshanshan*’ compression wood with a round shape, whose secondary walls are stained in different shades of red with saffron [62].

Generally, the reaction wood in coniferous trees is located on the lower side of an inclined trunk, and it experiences significant compressive stress; such wood is called compression wood (Figure 1b) [9,12]. However, Zajączkowska et al. found that compression wood does not always form on the same side of the trunk in their study of Norway spruce. Although compression wood initially forms in the lower side of the trunk, it sometimes appears on the upper side or the lateral side of the trunk over time [49]. The main difference between compression wood and normal wood lies in the structural differences in their tracheids [50]. In their longitudinal section, the shape of normal tracheids is usually very straight, but the tracheids in typical compression wood are noticeably shorter than those in normal wood [51,52], and their two ends form a cross-twisted shape, which is usually flat or has L-shaped needle tips [53] (Figure 6). In their transverse section, compression wood tracheids have circular or elliptical contours, and their inner walls are embedded with spiral cracks that penetrate deeply into the cell wall layer, with there being a large number of intercellular spaces between the tracheids [54,55,56] (Figure 7b), which are features that obviously contrast with the quadrilateral or hexagonal shape of normal wood tracheids (corresponding wood) [57] (Figure 7a). In addition, compared with normal wood, the diameter of compression wood tracheids is slightly smaller, but the thickness of their cell wall is significantly increased, leading to a corresponding decrease in the diameter of their lumen [58]. Liu et al. [59] studied the anatomical characteristics of compression wood under different inclination angles by conducting experiments on the tilted growth of *Pinus taeda* L.’s trunk. They found that as the inclination angle of the trunk increased, the intercellular spaces in the compression wood gradually increased, and the spiral cracks became more numerous, wider, and deeper.

Except for mild compression wood cells, the cell wall structure of compression wood cells often does not feature a S_3_ layer [51,52], and the microfibrillar angles of the S_2_ layer are relatively large [60]. In addition, there is a high density of lignin distribution in the S_2_ layer, which is an important feature of compression wood. Furthermore, the degree of cellulose polymerization and crystallinity in compression wood is lower than that in normal wood [61].

### 2.3. Reaction Tissues in Other Organs of Trees

**Figure 8:**
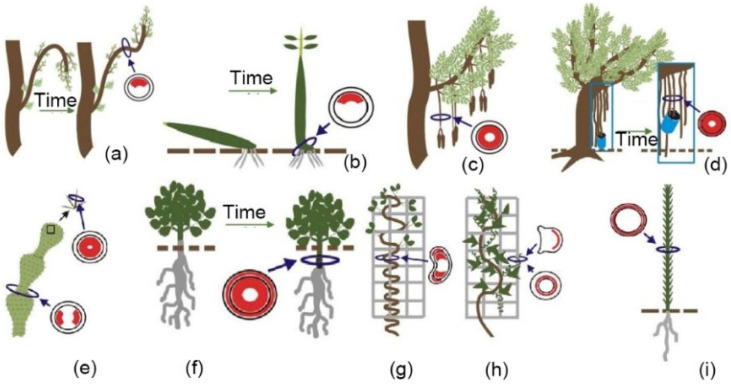
Distribution of reaction tissues (red areas) in various plants and organs [11]. (**a**) Branches of broad-leaved trees [68,69]; (**b**) stem of mangrove seedlings during gravitropic movement [70]; (**c**) pedicles of heavy fruits [63]; (**d**) Aerial roots [64]; (**e**) spines and cladode junction regions of *Opuntia* [71,72]; (**f**) roots and hypocotyls of geophytes [63,71]; (**g**,**h**) stems and tendrils of scandent and climbing plants [65,73]; (**i**) stems of fiber crops [64,72].

**Figure 9:**
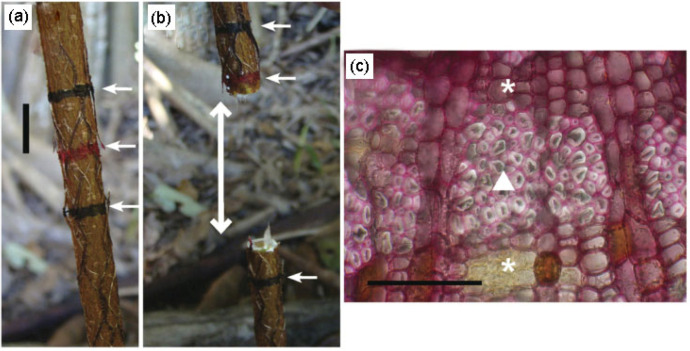
(**a**,**b**) Complete and severed grounded roots of *Ficus altissima*, with its cut surfaces separated by a distance of 3 cm (double-headed white arrow). Arrows indicate identifying marks [66]; (**c**) Secondary xylem of transected root * = parenchyma. ▲ = gelatinous fibers.

Reaction tissues not only exist in the trunks and branches of mature trees and their seedlings (Figure 8a,b), but also in some other tree organs. For example, the presence of gelatinous fibers has been found in the flower stalks of trees, this being an adaptive response to increases in fruit weight (Figure 8c). Sivan et al. [63] found that in *Couroupita*, the distribution of gelatinous fibers in different regions of the flower stalks was not uniform, which may be a response to different stress intensities in different regions of the stalks. As the fruit of a tree develops, the thickness of the gelatinous layer in the distal region of the fruit’s stalk, which is affected by the weight of the fruit, increases significantly. Gelatinous fibers also exist in the roots of trees. Zimmermann et al. [64] studied the anatomy of the aerial roots generated on the lower surface of the branches of *Ficus benjamina* L., and they found unlignified gelatinous fibers containing microfibrils that were almost parallel to the cell axis. These fiber cells were distributed symmetrically along the vertical axis in the aerial roots (Figure 8d). Further, when the aerial roots were buried in a pot, the researchers found that the contraction generated by the gelatinous fibers was strong enough to lift the pot off the ground (Figure 8d). In addition, after cutting the aerial roots of the *Ficus altissima*, they found that a contraction deformation up to 3 cm long occurred at the cutting site (Figure 8a,b) [65,66], with the gelatinous fibers in the roots being the driving force for this contraction (Figure 9c). Gelatinous fibers are also reported in normal roots, growing towards gravity in Acacia trees [67].

**Figure 10:**
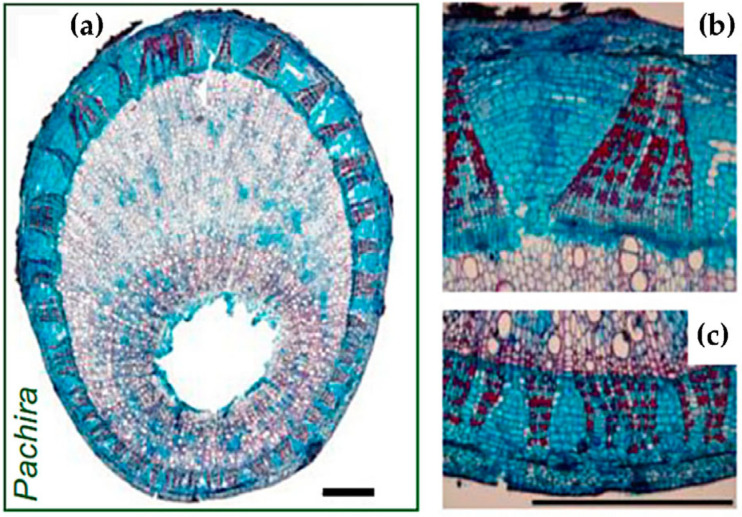
(**a**) Transverse sections of *Pachira aquatica* [74]; (**b**) upper side and (**c**) lower side of the tiled tree stem, including xylem and bark.

In addition, Clair et al. [74] found that the bark of tension wood is an important driver in the wood’s vertical growth against gravity. The researchers cultivated nine tropical tree species in tilted positions and observed that when external forces were released, the tilted trees tended towards upright growth, with their trunks bending into arcs. Among these species, five showed reduced curvature after some of their bark was removed, indicating the important role of bark in maintaining the vertical growth of trees. Further, through anatomical observations of bark and stem (Figure 10), the researchers found that its bark’s fibers have a trellis structure. As the tree grows, its bark’s circumference was found to increase, with the upper side of the bark of tension wood growing faster than other sides. Additionally, uneven tension in the trellis structure generates asymmetric forces, leading to the appearance of “anti-gravity” bending.

## 3. Reaction Tissues in Other Plants

In addition to trees, other plants develop specialized reaction tissues during their growth to maintain their growth status or adapt to their living environment. Researchers have made many important discoveries in the study of reaction tissues in various plants.

Gelatinous fibers, as an important feature of reaction tissues in broad-leaved plants, play an important biomechanical role in such organisms. In recent years, researchers have found that gelatinous fibers also exist in plants, including opuntia, scandent, and climbing plants. For example, Bobich and Nobel [71] found clusters of gelatinous fibers at the junctions of opuntia stems; these help to strengthen the connections between stems and resist lateral movement caused by environmental factors, such as wind (Figure 8e). Schreiber et al. [63] used Raman imaging to demonstrate that the shrinkage of the main roots of *Trifolium pratense* L. is due to the rich gelatinous layers in the plant’s cells, and that the gelatinous fibers are uniformly and symmetrically distributed in the transverse section of the roots (Figure 8f), with the fibers’ cell components and structures being very similar to those of tension wood in poplar. Tomlinson et al. [66] found gelatinous fibers in the roots of *Cycas* seedlings at different stages of its development, and they reported that the fibers’ structure is similar to that of tension wood in *Ficus*.

**Figure 11:**
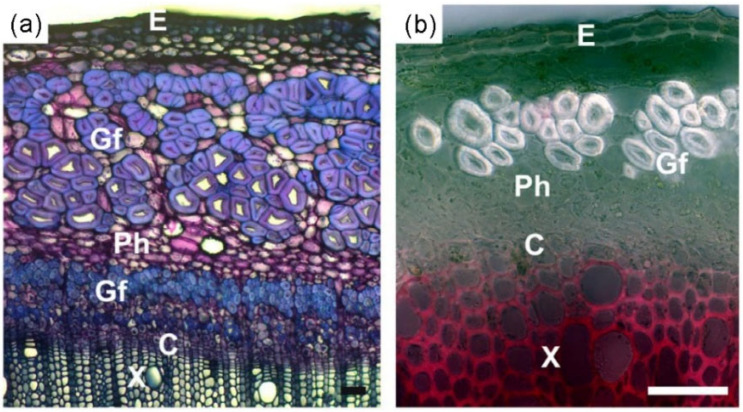
(**a**) Gelatinous fibers produced in primary and secondary phloem of *Cannabis sativa* stem and (**b**) phloem of *Linum usitatissimum* stem [11]. C: cambium; E: epidermis; Gf: gelatinous fibers; Ph: phloem; X: xylem.

Researchers have also found asymmetrically distributed gelatinous fibers in the xylem and bark of herbaceous and vine plants, with the content and distribution of gelatinous fibers found to vary among plant species. Figure 11 shows the distribution of gelatinous fibers in the bark of *Cannabis sativa* L. and *Linum usitatissimum* L. [11]. Fisher et al. [75] suggested that the large tensile stress and the tendency to contract produced by gelatinous fibers are the main factors causing the curling or winding growth of these plants (Figure 8g,h). Meloche et al. [65] also suggested that the tendrils of *redvine* use the contraction of gelatinous fibers to transform their long straight tendrils into highly curved structures. Furthermore, it has been found that the effect of gelatinous fibers in bark on the growth of vine stems is weaker than that in xylem [76].

**Figure 12:**
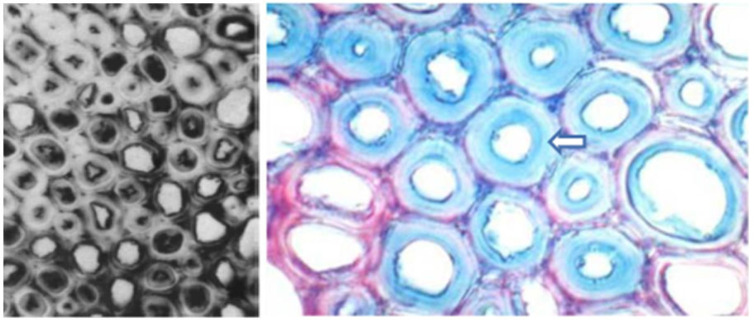
The gelatinous fibers of *Thyrsostachys oliveri* (the arrow indicates the gelatinous layer) [81].

Bamboo, as an important forest resource, has significant economic, social, and ecological value [77,78,79,80]. Therefore, the study of reaction tissues in bamboo is of great significance. As early as 1971, Liese and Grosser [81] attempted to find gelatinous fibers similar to tension wood in bamboo. They found the presence of a gelatinous layer in *Thyrsostachys oliveri* (Figure 12). However, due to a lack of understanding of the diversity of tension wood at that time, it was not comprehensive to judge whether it was reaction tissue based solely on the existence of the gelatinous layer. Recent studies have found that some broad-leaved trees have tension fibers with a multi-layered and alternating thin and thick cell wall structure similar to that found in bamboo fibers (Figure 5c), without the presence of a gelatinous layer [45,46]. Moreover, the arrangement of microfibrils in the cell walls of tension wood are almost parallel to the main axis, just like in bamboo fibers. From this perspective, bamboo as a whole resembles tension wood fibers to a certain extent. This can partly explain why slender bamboo has high toughness and tensile strength [82,83,84], which enables it to withstand external forces, such as wind, rain, and snow, with less tissue. These recent findings also provide inspiration and research directions for studying reaction tissues in bamboo.

## 4. Concluding Remarks

Reaction tissue in plants constitutes a response to plants’ own gravity or external forces. The result of long-term evolution, reaction tissue plays a decisive role in maintaining the shape and growth orientation of plants. In this study, the structural characteristics and biomechanical effects of reaction tissue in trees and other plants were summarized and analyzed. The cell wall structures of tension wood and compression wood have significant differences, with that of tension wood exhibiting greater diversity and variability. Gelatinous fibers are found in the reaction tissue in many plants and they play an important biomechanical role.

While the academic community has already continuously researched reaction tissue in trees, research on reaction tissue in other plants is gradually receiving widespread attention. Among such plants, bamboo, as an important non-wood material, the study of its special tissue structure is key to its rational processing and utilization. As mentioned above, research on the reaction tissue in bamboo is still stuck in the 1970s. Therefore, such research should look to develop new ideas, investigate the recent discoveries of tension wood with multi-layered cell wall structures, and draw lessons from research on reaction tissue in plants, such as vines and hemp, with extremely high length-to-diameter ratios in order to explore the relationship between the cell wall structure, components, and biomechanical effects of bamboo. The extensive exploration of the characteristics and properties of reaction tissues in various plants is of great significance for the study of biological structure and function, the development of new biomimetic materials, and the processing and utilization of plant materials. In addition, research on plant reaction tissues should combine research on anatomy, chemical composition, hormone distribution, mechanical properties, and genes, and explore the adaptive responses of plants to themselves or the environment from the perspectives of multiple disciplinary fields, such as biomechanics, structural botany, plant chemistry, and plant physiology.

## Data Availability

No new data were created or analyzed in this study. Data sharing is not applicable to this article.
